# Effects of high-intensity interval training on vascular function in patients with cardiovascular disease: a systematic review and meta-analysis

**DOI:** 10.3389/fphys.2023.1196665

**Published:** 2023-07-27

**Authors:** Laura Fuertes-Kenneally, Carles Blasco-Peris, Antonio Casanova-Lizón, Sabina Baladzhaeva, Vicente Climent, José Manuel Sarabia, Agustín Manresa-Rocamora

**Affiliations:** ^1^ Alicante Institute for Health and Biomedical Research (ISABIAL), Alicante, Spain; ^2^ Dr. Balmis General University Hospital, Alicante, Spain; ^3^ Department of Physical Education and Sport, University of Valencia, Valencia, Spain; ^4^ Department of Sport Sciences, Miguel Hernández University of Elche, Elche, Spain

**Keywords:** high-intensity interval training, vascular function, cardiovascular disease, training variables, flow-mediated dilation, endothelial progenitor cells, coronary artery disease, heart failure

## Abstract

**Background:** Exercise training improves endothelial function in patients with cardiovascular disease (CVD). However, the influence of training variables remains unclear. The aim of this study was to evaluate the effect of high-intensity interval training (HIIT), compared to moderate intensity training (MIT) and other exercise modalities (i.e., resistance and combined exercise), on endothelial function, assessed by arterial flow-mediated dilation (FMD) or endothelial progenitor cells (EPCs), in patients with CVD. Secondly, we investigated the influence of other training variables (i.e., HIIT protocol).

**Methods:** The PICOS strategy was used to identify randomised and non-randomised studies comparing the effect of HIIT and other exercise modalities (e.g., MIT) on endothelial function in patients with CVD. Electronic searches were carried out in Pubmed, Embase, and Web of Science up to November 2022. The TESTEX scale was used to evaluate the methodological quality of the included studies. Random-effects models of between-group mean difference (MD) were estimated. A positive MD indicated an effect in favour of HIIT. Heterogeneity analyses were performed by the chi-square test and *I* ^*2*^ index. Subgroup analyses evaluated the influence of potential moderator variables.

**Results:** Fourteen studies (13; 92.9% randomised) were included. Most of the studies trained 3 days a week for 12 weeks and performed long HIIT. No statistically significant differences were found between HIIT and MIT for improving brachial FMD in patients with coronary artery disease (CAD) and heart failure with reduced ejection fraction (HFrEF) (8 studies; MD_+_ = 0.91% [95% confidence interval (CI) = −0.06, 1.88]). However, subgroup analyses showed that long HIIT (i.e., > 1 min) is better than MIT for enhancing FMD (5 studies; MD_+_ = 1.46% [95% CI = 0.35, 2.57]), while no differences were found between short HIIT (i.e., ≤ 1 min) and MIT (3 studies; MD_+_ = −0.41% [95% CI = −1.64, 0.82]). Insufficient data prevented pooled analysis for EPCs, and individual studies failed to find statistically significant differences (*p* > .050) between HIIT and other exercise modalities in increasing EPCs.

**Discussion:** Poor methodological quality could limit the precision of the current results and increase the inconsistency. Long HIIT is superior to MIT for improving FMD in patients with CAD or HFrEF. Future studies comparing HIIT to other exercise modalities, as well as the effect on EPCs and in HF with preserved ejection fraction are required.

**Systematic Review Registration:** https://www.crd.york.ac.uk/PROSPERO/#myprospero, identifier CRD42022358156.

## 1 Introduction

Endothelial function is defined as the ability of the endothelium to produce vasodilators that control vascular tone, blood flow, and immune cell activity (Godo and Shimokawa, 2017). The most frequently used technique to assess endothelial function is flow-mediated dilation (FMD), which is largely caused by the release of endothelium-derived relaxing factors (e.g., nitric oxide [NO]) (Koller et al., 1995). Brachial FMD is the dilation of the artery induced by a reactive hyperaemia after vessel occlusion and release (Korkmaz and Onalan, 2008). Another variable used to evaluate endothelial function are endothelial progenitor cells (EPCs) (Hill et al., 2003). There are a small population of bone marrow-derived cells which are mobilised to the peripheral blood by various stimuli such as ischemia or chemokines and participate in endothelial repairing and neovascularisation (Khakoo and Finkel, 2005). There is evidence that patients with cardiovascular disease (CVD) (e.g., coronary artery disease [CAD], heart failure with reduced ejection fraction [HFrEF], and heart failure with preserved ejection fraction [HFpEF]), suffer from endothelial dysfunction, which may partly explain the increased risk of mortality found in this population (Katz et al., 2005). Therefore, there is a growing interest in the development of pharmacological and non-pharmacological treatments capable of improving endothelial function (i.e., FMD and EPCs) and reducing mortality risk of patients with CVD (Medina-Leyte et al., 2021).

Regarding non-pharmacological interventions, exercise-based cardiac rehabilitation (CR) has been demonstrated to be effective for the prevention and treatment of CVD by improving endothelial dysfunction (Pearson and Smart, 2017a; 2017b; Sakellariou et al., 2021; Manresa-Rocamora et al., 2022). Nonetheless, exercise training variables (e.g., exercise modality [e.g., aerobic exercise and resistance exercise], training frequency, and intervention length) should be taken into account to properly design CR programmes aimed at improving endothelial function (Sarabia et al., 2018). Aerobic exercise is the most common exercise modality used in exercise-based CR programmes (Anderson et al., 2016). Within aerobic exercise, moderate intensity training (MIT) works in the treatment of patients with CVD (Manresa-Rocamora et al., 2020; Manresa-Rocamora et al., 2021). MIT is conducted over a long period of time (e.g., 30—40 min) at moderate intensity (e.g., between first and second ventilatory thresholds) (Fletcher et al., 2001). Nonetheless, high-intensity interval training (HIIT) has emerged as a more effective and time-efficient exercise modality in the treatment of patients with CVD (Gillen and Gibala, 2018). HIIT combines high-intensity bouts of exercise with periods of passive rest or low-intensity exercise (Buchheit and Laursen, 2013a; Buchheit and Laursen, 2013b).

Ramos et al. (2015) and Mattioni Maturana et al. (2021) showed that HIIT increases endothelial function (i.e., FMD) to a greater degree than MIT in healthy individuals and patients with a wide range of pathologies. In the same line, Fuertes-Kenneally et al. (2023) found that HIIT is superior to MIT for enhancing FMD in patients with HFrEF. Nonetheless, few studies were included, and their findings were inconsistent. In this regard, prescription of HIIT requires the manipulation of variables such as duration of high-intensity exercise bouts (i.e., HIIT protocol) (e.g., > 1 min [long HIIT] or ≤ 1 min [short HIIT]), number of repetitions and series, type of recovery (i.e., active or passive), and duration of the recovery periods (Buchheit and Laursen, 2013a; Buchheit and Laursen, 2013b). The manipulation of any of these variables can affect the physiological response to HIIT (Buchheit and Laursen, 2013a; Buchheit and Laursen, 2013b). However, previous meta-analyses have not addressed whether variables such as number of repetitions, duration of high-intensity exercise sessions, or type of recovery (i.e., HIIT variables) can affect the HIIT-induced effect on endothelial function in patients with CVD. On the other hand, resistance exercise, which can be performed alone or combined with aerobic exercise (henceforth combined exercise), acutely increases EPCs in healthy women (Ribeiro et al., 2017). Nonetheless, whether HIIT is superior to resistance exercise or combined exercise in patients with CVD remains unclear.

Therefore, the aims of the current study were the following: (a) to systematically compile training characteristics of studies that investigated the effect of HIIT, compared to MIT and other exercise modalities (i.e., resistance exercise and combined exercise), on endothelial function (i.e., brachial FMD and EPCs) in patients with CVD (i.e., CAD, HFrEF, and HFpEF); (b) to determine whether HIIT improves endothelial function to a greater extent than other exercise interventions; (c) to study the influence of HIIT variables on the improvement of endothelial function in patients with CVD.

## 2 Method

This systematic review with meta-analysis was conducted in accordance with the Preferred Reporting Items for Systematic Reviews and Meta-analysis (PRISMA) guidelines (Page et al., 2021) and was prospectively registered on the International Prospective Register of Systematic Reviews (PROSPERO) database prior to formal screening (CRD42022358156).

### 2.1 Data search and sources

An electronic search strategy was specifically designed for Pubmed, Embase, Cochrane Central database, and Web of Science (i.e., Core Collection [Science Citation Index Expanded and Conference Proceeding Citation Index]). Free-text terms, which were searched in the title, abstract, and keywords (where available) fields, and thesaurus terms (e.g., Medical Subject Heading [MeSH]) were included in the search strategy. Database searches were performed from earliest record to November 2022. The PIO strategy (participants [e.g., CVD], interventions [e.g., HIIT], and outcomes [e.g., FMD]) was used to create the structure of the bibliographic search (da Costa Santos et al., 2007). Full database searches can be found in Supplementary Material (SM). Moreover, the reference lists of the included articles were hand-reviewed using the backward reference searching to identify further published studies (Horsley et al., 2011). Finally, corresponding authors of the selected studies were emailed in an attempt to retrieve published, unpublished, and ongoing studies. Email contact was maintained with these authors to identify changes in the status of these studies.

### 2.2 Study selection

Eligibility criteria were established according to the PICOS guideline (participants, interventions, comparisons, outcomes, and study design) as follows: (a) participants: adult male and female patients with CVD (i.e., CAD and HFrEF, and HFpEF); (b) interventions: inpatient or outpatient HIIT programmes lasting at least 2 weeks, regardless of the setting (i.e., home- or centre-based CR programme), either alone or in addition to psychosocial and/or educational interventions; (c) comparison groups (CG): non-exercise group, MIT, resistance exercise, and combined aerobic and resistance exercise [(henceforth combined exercise)]; (d) outcomes: endothelial function assessed by brachial FMD and reported as percent change and/or circulating blood markers (i.e., EPCs); and (e) study design: randomised and non-randomised studies. Finally, studies written in English, Spanish, or Russian were included. When several articles referred to the same study, the original article was included in the review.

Two review authors (A.C. and S.B) independently evaluated each study for inclusion in two stages: (a) a preliminary assessment of the titles/abstracts and (b) a thorough review of the full-text studies selected in the previous stage. In case of disagreements, a third author (A.M.) checked the study information to reach an agreement.

### 2.3 Data extraction and coding study characteristics

The data were extracted using a standardised extraction form in Microsoft Excel by two review authors (A.C. and S.B.). In case of doubts, a third author (A.M.) assessed the information to reach an agreement. The following information was extracted from the selected studies: (a) study information (i.e., publication year, country, journal, sample size, and study design [i.e., randomised or non-randomised]; (b) study population (i.e., clinical condition [i.e., CAD, HFrEF, and HFpEF], sex [i.e., males, females, or mixed sample], men percentage, age, baseline artery diameter [mm], and VO_2_ peak); (c) intervention (i.e., CR phase [i.e., inpatient or outpatient], setting [i.e., home-based, centre-based, or mixed programme], exercise mode [e.g., cycle ergometer], number of bouts, duration of high-intensity intervals [i.e., long HIIT (>60 s) or short HIIT (≤60 s)], intensity, type of recovery [i.e., active or passive], recovery length, and recovery intensity [if applicable]); (d) CG characteristics (e.g., exercise modality [i.e., MIT, resistance exercise, or combined exercise], session length, intensity, and number of resistance exercises); (e) FMD assessment characteristics (cuff placement [i.e., distal or proximal to the imaged artery], occlusion length [s], occlusion pressure [mmHg], and post-deflation time window [s]); (f) EPCs assessment (i.e., method used to enumerate circulating EPCs, antibodies used, EPCs phenotype, and units of measure); and (g) statistical information (e.g., mean and standard deviation [*SD*]).

### 2.4 Dealing with missing data

Corresponding authors were emailed to request missing data (e.g., number of patients allocated to the two groups, training frequency, and endpoint information). Authors of unpublished studies (e.g., conference abstract) were also contacted to request information not reported in the abstract. If no answer was received, the unpublished study was excluded from the review.

### 2.5 Methodological quality assessment

The tool for the assessment of study quality and reporting in exercise (TESTEX) scale was used to carry out the methodological quality judgement of the included studies (Smart et al., 2015). The criteria used to carry out methodological quality assessment can be found in Table S1 (see SM). Based on the overall scores, methodological quality was judged as excellent (12—14), good (9—11), fair (6—8), or poor (<6). Two authors (C.B. and L.F.) carried out the methodological quality assessment and, in case of doubts, a third author (A.M.) checked the specific item to reach an agreement.

### 2.6 Computation of effect size and statistical analyses

The mean difference (MD) with its 95% confidence interval (CI) was used as the effect size (ES) index, as the eligible studies had to measure relative changes in brachial FMD (inclusion criteria) (Andrade, 2020). The MD was calculated by subtracting the mean change in the CG (i.e., MIT, resistance exercise, or combined exercise) from the mean change in the HIIT group. Later, MD was corrected by a factor for small samples (Hedges and Olkin, 2014). Means and *SD* were estimated from reported data (e.g., median, interquartile range, and group sample size) where necessary (Wan et al., 2014). In multistage studies (i.e., more than two assessments), the results obtained from the first two measurements were used to calculate the MD. Separate meta-analyses were performed based on the CG. Moreover, it was established that at least three studies must have examined the effect of HIIT on vascular function on a CVD sublevel (i.e., CAD, HFrEF, or HFpEF) to include this pathology in pooled analyses. Afterwards, outcomes common to at least three studies were pooled using random-effects meta-analyses (Borenstein et al., 2010). The results of the non-pooled studies were discussed qualitatively.

Sensitivity analyses were performed by applying the leave-one-out cross-validation method to test the influence of each individual study in our pooled results. Studies were considered influential if their removal significantly changed the pooled effect (e.g., change from significant to non-significant). After deleting influential studies from the pooled finding (if necessary), the chi-square test and *I* ^*2*^ index were used to analyse heterogeneity. *I* ^*2*^ values of 25%, 50%, and 75% were interpreted as low, moderate, and high heterogeneity, respectively (Higgins et al., 2003). In case of substantial heterogeneity (i.e., *p* ≤ .050 and/or *I* ^*2*^ ≥ 50%), the influence of potential moderator variables of the pooled finding was investigated (Higgins et al., 2003). Subgroup analyses were used to carry out heterogeneity analyses. All analyses were performed using weighted least squares and assuming mixed-effect models (Cooper and Hedges, 1994). Publication bias was analysed graphically through contour-enhanced funnel plots, while the Egger’s test was used to quantify the evidence for funnel plot asymmetry (Peters et al., 2008; Sterne et al., 2011). STATA software (version 16.0; Stata Corp LLC, College Station, TX, United States) was used to conduct all analyses.

## 3 Results

### 3.1 Study selection

**FIGURE 1:**
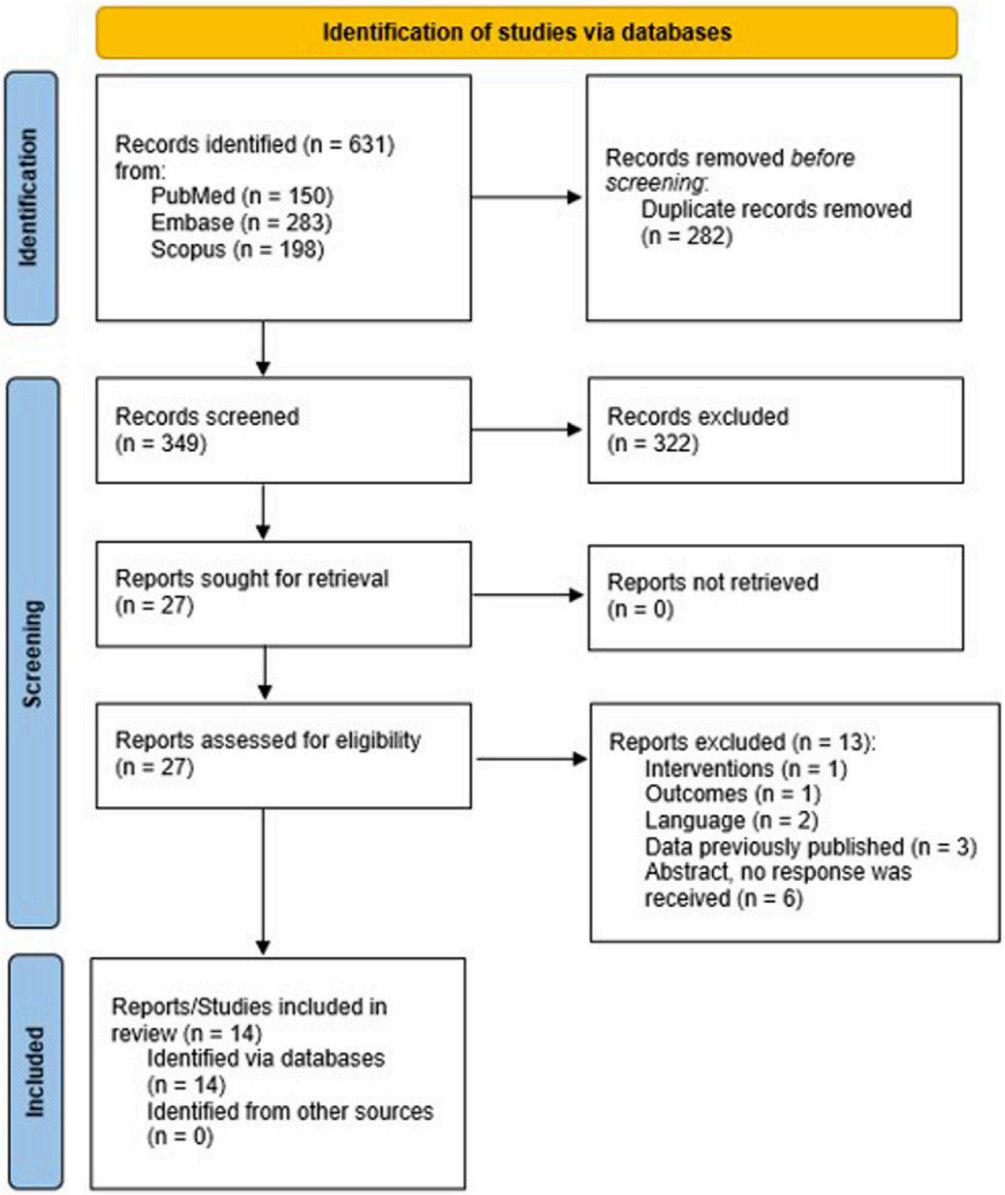
Flow-chart of the study selection process.

Figure 1 shows the flow chart diagram of the study selection process, according to the PRISMA guidelines. A total of 631 references were retrieved from the electronic searches. Two hundred and eighty-two duplicates were removed, and 349 references were forwarded to the first stage of the study selection process. After title/abstract review, 322 studies were excluded and 27 were full-text checked against inclusion criteria (second stage). Fourteen studies met the inclusion criteria (Wisløff et al., 2007; Anagnostakou et al., 2011; Moholdt et al., 2012; Currie et al., 2013; Suchy et al., 2014; Angadi et al., 2015; Benda et al., 2015; Van Craenenbroeck et al., 2015; Zaky et al., 2018; Sales et al., 2020; Kourek et al., 2021; Turri-Silva et al., 2021; Taylor et al., 2022; Valentino et al., 2022) and 13 were excluded from qualitative synthesis for the following reasons: (a) interventions (*n* = 1) (Deljanin Ilic et al., 2009); (b) outcomes (*n* = 1) (Tryfonos et al., 2021); (c) language (*n* = 2) (Nechwatal et al., 2002; Nik-Maleki et al., 2018); (d) data previously published (*n* = 3) (Conraads et al., 2015; Pattyn et al., 2016; Thijssen et al., 2019); and (e) unpublished study with no author response (*n* = 6).

### 3.2 Study characteristics

The main characteristics of the included studies are delivered in Table 1. The studies were published from 2007 to 2022 (Wisløff et al., 2007; Anagnostakou et al., 2011; Moholdt et al., 2012; Currie et al., 2013; Suchy et al., 2014; Angadi et al., 2015; Benda et al., 2015; Van Craenenbroeck et al., 2015; Zaky et al., 2018; Sales et al., 2020; Kourek et al., 2021; Turri-Silva et al., 2021; Taylor et al., 2022; Valentino et al., 2022). The endothelial function results from the study conducted by Suchy et al. (2014) were not available at the time of our data search. However, we reached out to the authors to obtain their unpublished data, in order to include it in our meta-analysis. Subsequently, these results were published in a separate article (Gevaert et al., 2023) after we had completed our review. Therefore, we deemed it more appropriate to cite both the original study and the subsequent publication (Suchy et al., 2014; Gevaert et al., 2023). Out of all the 14 studies, 13 (92.9%) were randomised (Wisløff et al., 2007; Anagnostakou et al., 2011; Moholdt et al., 2012; Currie et al., 2013; Suchy et al., 2014; Angadi et al., 2015; Van Craenenbroeck et al., 2015; Zaky et al., 2018; Sales et al., 2020; Kourek et al., 2021; Turri-Silva et al., 2021; Taylor et al., 2022; Valentino et al., 2022) and one (7.1%) was non-randomised (Benda et al., 2015). Thirteen studies (92.9%) recruited male and female patients (Wisløff et al., 2007; Anagnostakou et al., 2011; Moholdt et al., 2012; Currie et al., 2013; Suchy et al., 2014; Angadi et al., 2015; Benda et al., 2015; Van Craenenbroeck et al., 2015; Sales et al., 2020; Kourek et al., 2021; Turri-Silva et al., 2021; Taylor et al., 2022; Valentino et al., 2022), and one (7.1%) included exclusively male patients (Zaky et al., 2018). Five studies (35.7%) included patients with CAD (Moholdt et al., 2012; Currie et al., 2013; Van Craenenbroeck et al., 2015; Taylor et al., 2022; Valentino et al., 2022), six (42.9%) patients with HFrEF (Wisløff et al., 2007; Anagnostakou et al., 2011; Benda et al., 2015; Zaky et al., 2018; Sales et al., 2020; Kourek et al., 2021), two (14.3%) patients with HFpEF (Suchy et al., 2014; Angadi et al., 2015), and one (7.1%) recruited both patients with HFrEF and patients with HFpEF (Turri-Silva et al., 2021). Eleven studies (78.6%) defined a MIT group (Wisløff et al., 2007; Moholdt et al., 2012; Currie et al., 2013; Suchy et al., 2014; Angadi et al., 2015; Benda et al., 2015; Van Craenenbroeck et al., 2015; Zaky et al., 2018; Sales et al., 2020; Taylor et al., 2022; Valentino et al., 2022), one (7.1%) a resistance exercise group (Turri-Silva et al., 2021), and two (14.3%) a combined exercise group (Anagnostakou et al., 2011; Kourek et al., 2021). There were 309 patients who completed the intervention in the HIIT groups (min—max sample size: 5—85 patients) and 339 in the CG (min—max sample size: 6—89 patients). Among studies with available information, the mean male percentage in the HIIT groups and CG was 73.8% (*n* = 13) and 73.7% (*n* = 11), respectively. In the HIIT groups and CG, the mean ± *SD* age was 61.3 ± 7.0 years (*n* = 14; min—max: 52.0—76.5 years) and 62.0 ± 6.8 years (*n* = 14; min—max: 52.8—74.4 years), and the mean ± *SD* VO_2_ peak was 20.3 ± 4.9 mL·kg^−1^·min^−1^ (n = 13; min—max: 13.0—31.6 mL·kg^−1^·min^−1^) and 20.0 ± 5.2 mL·kg^−1^·min^−1^ (n = 13; min—max: 13.0—32.2 mL·kg^−1^·min^−1^), respectively.

**TABLE 1:** Study and patient characteristics.

Study		Group	Study characteristics	Patient characteristics			
			Country; study design; journal	Pathology; sample size; male percentage; age		Baseline brachial diameter; LVEF; VO_2_ peak	
Anagnostakou et al. (2011)		HIIT	Greece; randomised; J Card Fail	HFrEF; 14 (14); 86%; 52.0 ± 11.0 years		4.40 ± 0.50 mm; 36.0% ± 13.0%; 15.7 ± 4.0 mL·kg^−1^·min^−1^	
		Combined exercise		HFrEF; 14 (14); 79%; 54.0 ± 10.0 years		4.50 ± 0.50 mm; 39.0% ± 11.0%; 15.7 ± 6.0 mL·kg^−1^·min^−1^	
Angadi et al. (2015)		HIIT	United States; randomised; J Appl Physiol	HFpEF; 9 (9); 89%; 69.0 ± 6.1 years		NR; 65.0% ± 5.0%; 19.2 ± 5.2 mL·kg^−1^·min^−1^	
		MIT		HFpEF; 6 (6); 67%; 71.5 ± 11.7 years		NR; 66.0% ± 4.0%; 16.9 ± 3.0 mL·kg^−1^·min^−1^	
Benda et al. (2015)		HIIT	Netherlands; non-randomised; PloS One	HFrEF; 10 (10); 90%; 63.0 ± 8.0 years		4.40 ± 0.90 mm; 37.0% ± 6.0%; 19.1 ± 4.1 mL·kg^−1^·min^−1^	
		MIT		HFrEF; 10 (10); 100%; 64.0 ± 8.0 years		4.50 ± 0.50 mm; 38.0% ± 6.0%; 21.0 ± 3.4 mL·kg^−1^·min^−1^	
Currie et al. (2013)		HIIT	Canada; randomised; Med Sci Sports Exerc	CAD; 11 (11); NR; 62.0 ± 11.0 years		4.52 ± 0.70 mm; 28.0% ± 7.0%; 19.8 ± 3.7 mL·kg^−1^·min^−1^	
		MIT		CAD; 11 (11); NR; 68.0 ± 8.0 years		4.30 ± 0.75 mm; 30.0% ± 5.7%; 18.7 ± 5.7 mL·kg^−1^·min^−1^	
Kourek et al. (2021)		HIIT	Grece; randomised; Int J Cardiol Heart Vasc	HFrEF; 21 (21); 81%; 55.0 ± 11.0 years		NR; 35% (30—43) ^#^; 18.7 ± 5.0 mL·kg^−1^·min^−1^	
		Combined exercise		HFrEF; 23 (23); 78%; 57.0 ± 9.0 years		NR; 30% (25—35) ^#^; 18.2 ± 3.8 mL·kg^−1^·min^−1^	
Moholdt et al. (2012)		HIIT	Norway; randomised; Clin Rehabil	CAD; 30 (24); 83%; 56.7 ± 10.4 years		NR; NR; 32.2 ± 6.7 mL·kg^−1^·min^−1^	
		MIT		CAD; 59 (44); 83%; 57.7 ± 9.3 years		NR; NR; 16.5 ± 2.7 mL·kg^−1^·min^−1^	
Sales et al. (2020)		HIIT	Brazil; randomised; Circ Heart Fail	HFrEF; 11 (9); 64%; 55.0 ± 7.6 years		4.20 ± 0.50 mm; 27.8% ± 9.5%; 17.9 ± 3.2 mL·kg^−1^·min^−1^	
		MIT		HFrEF; 11 (10); 64%; 59.5 ± 7.0 years		4.00 ± 0.80 mm; 31.3% ± 6.1%; 16.9 ± 1.9 mL·kg^−1^·min^−1^	
Suchy et al. (2014)/Gevaert et al. (2023)		HIIT	Germany; randomised; Eur J Prev Cardiol/JACC Heart Fail	HFpEF; 58 (15)*; 29%; 70.0 ± 7.0 years		NR; 50% or greater (IC); 18.9 ± 5.4 mL·kg^−1^·min^−1^	
		MIT		HFpEF; 58 (18)*; 40%; 70.0 ± 8.0 years		NR; 50% or greater (IC); 18.2 ± 5.1 mL·kg^−1^·min^−1^	
Taylor et al. (2022)		HIIT	Australia; randomised; Scand J Med Sci Sports	CAD: 27 (19); 95%; 65.0 ± 7.0 years		0.46 ± 0.07 mm; NR; 27.9 ± 6.4 mL·kg^−1^·min^−1^	
		MIT		CAD; 25 (16); 88%; 63.0 ± 8.0 years		0.46 ± 0.05 mm; NR; 27.2 ± 7.3 mL·kg^−1^·min^−1^	
Turri-Silva et al. (2021)		HIIT	Brazil; randomised; PloS One	HFrEF and HFpEF; 8 (5); 63%; 60.9 ± 9.7 years		4.54 ± 1.09 mm; 50.4% ± 17.0%; 17.5 ± 4.2 mL·kg^−1^·min^−1^	
		Resistance exercise		HFrEF and HFpEF; 6 (6); 67%; 55.0 ± 10.9 years		4.38 ± 0.58 mm; 42.2% ± 13.5%; 16.9 ± 2.5 mL·kg^−1^·min^−1^	
Valentino et al. (2022)		HIIT	Canada; randomised; Physiol Rep	CAD; 9 (9); 89%; 62.8 ± 6.1 years		4.60 ± 0.70 mm; NR; 21.4 ± 4.5 mL·kg^−1^·min^−1^	
		MIT		CAD; 9 (9); 89%; 60.0 ± 9.0 years		4.00 ± 0.60 mm; NR; 22.9 ± 2.5 mL·kg^−1^·min^−1^	
Van Craenenbroeck et al. (2015)		HIIT	Belgium; randomised; Am J Physiol Heart Circ Physiol	CAD; 85 (76); 91%; 57.0 ± 8.8 years		4.00 ± 0.60 mm; 57.1% ± 8.5%; 23.3 ± 5.8 mL·kg^−1^·min^−1^	
		MIT		CAD; 89 (84); 89%; 59.9 ± 9.2 years		3.90 ± 0.60 mm; 56.8% ± 7.7%; 22.2 ± 5.6 mL·kg^−1^·min^−1^	
Wisløff et al. (2007)		HIIT	Norway; randomised; Circulation	HFrEF; 9 (9); 78%; 76.5 ± 9.0 years		NR; 28.0% ± 7.3%; 13.0 ± 1.6 mL·kg^−1^·min^−1^	
		MIT		HFrEF; 8 (8); 78%; 74.4 ± 12.0 years		NR; 32.8% ± 4.8%; 13.0 ± 1.1 mL·kg^−1^·min^−1^	
Zaky et al. (2018)		HIIT	Egypt; randomised; Biosci Res	HFrEF; 20 (20); 100%; 54.0 ± 2.7 years		4.13 ± 0.26 mm; 37.0% ± 1.9%; NR	
		MIT		HFrEF; 20 (20); 100%; 52.8 ± 11.6 years		4.13 ± 0.27 mm; 37.5% ± 3.1%; NR	

CAD, coronary artery disease; IC, inclusion criterion; HFpEF, heart failure with preserved ejection fraction; HFrEF, heart failure with reduced ejection fraction; HIIT, high-intensity interval training; LVEF, left ventricular ejection fraction; MIT, moderate intensity training; NR, not reported; VO_2_ peak, peak oxygen uptake.

Values are reported as mean ± standard deviation, unless otherwise stated; # Data expressed as median (25th—75th percentiles); sample size in brackets refers to the number of patients included in the analysis; * The number of patients with available data on endothelial progenitor cells is different.

The main characteristics of the HIIT groups and CG can be found in Supplementary Table S2. Out of the 14 studies, three (21.4%) were multistage and several measurements were conducted (Suchy et al., 2014; Taylor et al., 2022; Valentino et al., 2022). The following characteristics refer to the first stage. All included studies conducted an outpatient exercise-based CR programme (Wisløff et al., 2007; Anagnostakou et al., 2011; Moholdt et al., 2012; Currie et al., 2013; Suchy et al., 2014; Angadi et al., 2015; Benda et al., 2015; Van Craenenbroeck et al., 2015; Zaky et al., 2018; Sales et al., 2020; Kourek et al., 2021; Turri-Silva et al., 2021; Taylor et al., 2022; Valentino et al., 2022). Nine studies (64.3%) carried out centre-based exercise sessions (Anagnostakou et al., 2011; Angadi et al., 2015; Benda et al., 2015; Van Craenenbroeck et al., 2015; Zaky et al., 2018; Sales et al., 2020; Kourek et al., 2021; Turri-Silva et al., 2021; Valentino et al., 2022) and four (35.7%) combined centre and home-based exercise sessions (Wisløff et al., 2007; Moholdt et al., 2012; Currie et al., 2013; Taylor et al., 2022), while one study (7.1%) carry out exclusively supervised exercise sessions in the HIIT group and added unsupervised sessions in the CG (i.e., centre and home-based exercise sessions) (Suchy et al., 2014). Eleven studies (78.6%) were of a 12-week duration (Wisløff et al., 2007; Anagnostakou et al., 2011; Moholdt et al., 2012; Currie et al., 2013; Suchy et al., 2014; Benda et al., 2015; Van Craenenbroeck et al., 2015; Zaky et al., 2018; Sales et al., 2020; Kourek et al., 2021; Turri-Silva et al., 2021) and three (21.4%) of a 4-week duration (Angadi et al., 2015; Taylor et al., 2022; Valentino et al., 2022). Eleven studies (78.7%) carried out three sessions a week (Wisløff et al., 2007; Anagnostakou et al., 2011; Moholdt et al., 2012; Currie et al., 2013; Angadi et al., 2015; Van Craenenbroeck et al., 2015; Zaky et al., 2018; Sales et al., 2020; Kourek et al., 2021; Turri-Silva et al., 2021; Taylor et al., 2022), one (7.1%) trained twice a week (Benda et al., 2015), one (7.1%) performed different number of exercise sessions a week in the HIIT group (i.e., 3 sessions) and CG (i.e., 5 sessions) (Suchy et al., 2014), and one (7.1%) did not report this information (Valentino et al., 2022). Training sessions were mainly performed on a cycle ergometer (eight studies; 57.1%) or on a treadmill (three studies; 21.4%). Regarding HIIT interventions, five studies (35.7%) performed short HIIT (≤1 min) (Anagnostakou et al., 2011; Currie et al., 2013; Benda et al., 2015; Zaky et al., 2018; Valentino et al., 2022) and nine (64.3%) long HIIT (>1 min) (Wisløff et al., 2007; Moholdt et al., 2012; Suchy et al., 2014; Angadi et al., 2015; Van Craenenbroeck et al., 2015; Sales et al., 2020; Kourek et al., 2021; Turri-Silva et al., 2021; Taylor et al., 2022), of which, seven studies (77.8%) carried out four 4-min high-intensity intervals (Wisløff et al., 2007; Anagnostakou et al., 2011; Moholdt et al., 2012; Suchy et al., 2014; Angadi et al., 2015; Van Craenenbroeck et al., 2015; Kourek et al., 2021; Taylor et al., 2022). Out of the 14 studies included, 13 (92.9%) performed an active recovery period between high-intensity intervals (Wisløff et al., 2007; Moholdt et al., 2012; Currie et al., 2013; Suchy et al., 2014; Angadi et al., 2015; Benda et al., 2015; Van Craenenbroeck et al., 2015; Zaky et al., 2018; Sales et al., 2020; Kourek et al., 2021; Turri-Silva et al., 2021; Taylor et al., 2022; Valentino et al., 2022) and one (7.1%) a passive recovery (Anagnostakou et al., 2011). The length of the recovery period ranged from 60 s to 240 s.

Regarding the outcomes reported, 11 studies (78.6%) reported brachial FMD (Wisløff et al., 2007; Anagnostakou et al., 2011; Moholdt et al., 2012; Currie et al., 2013; Angadi et al., 2015; Benda et al., 2015; Zaky et al., 2018; Sales et al., 2020; Turri-Silva et al., 2021; Taylor et al., 2022; Valentino et al., 2022), two (14.3%) brachial FMD and EPCs (Suchy et al., 2014; Van Craenenbroeck et al., 2015), and one (7.1%) EPCs (Kourek et al., 2021). The characteristics of endothelial function assessment can be found in Supplementary Table S2. Briefly, out of the 13 studies which assessed brachial FMD, six (46.2%) put the bracelet distal to the brachial artery (i.e., forearm) (Anagnostakou et al., 2011; Currie et al., 2013; Benda et al., 2015; Van Craenenbroeck et al., 2015; Sales et al., 2020; Valentino et al., 2022), three (23.0%) proximal (i.e., upper-arm) (Moholdt et al., 2012; Zaky et al., 2018; Turri-Silva et al., 2021), and four (30.8%) did not explicitly report this information (Wisløff et al., 2007; Suchy et al., 2014; Angadi et al., 2015; Taylor et al., 2022). Among the studies with available information, six (66.7%) carried out continuous measurements after cuff deflation (Anagnostakou et al., 2011; Currie et al., 2013; Benda et al., 2015; Van Craenenbroeck et al., 2015; Turri-Silva et al., 2021; Valentino et al., 2022) and three (33.3%) conducted specific measurements over time (Wisløff et al., 2007; Moholdt et al., 2012; Zaky et al., 2018). The three studies which assessed EPCs used flow cytometry (Suchy et al., 2014; Van Craenenbroeck et al., 2015; Kourek et al., 2021). Van Craenenbroeck et al. (2015) defined EPCs as CD34^+^/KDR^+^/CD45_dim_ and Suchy et al. (2014) as CD45^dim^/CD34^+^/VEGFR_2_^+^. Kourek et al. (2021) defined three EPCs subgroups as following: (a) CD34^+^/CD45^-^/CD133^+^; (b) CD34^+^/CD45^-^/CD133^+^/VEGFR_2_, and (c) CD34^+^/CD133^-^/VEGFR_2_.

### 3.3 Methodological quality assessment

The results of the assessment of the methodological quality can be found in Table 2. Two studies (14.3%) were classified as good methodological quality (Moholdt et al., 2012; Valentino et al., 2022), seven (50.0%) as fair (Wisløff et al., 2007; Anagnostakou et al., 2011; Suchy et al., 2014; Benda et al., 2015; Van Craenenbroeck et al., 2015; Turri-Silva et al., 2021; Taylor et al., 2022), and five (35.7%) as poor (Currie et al., 2013; Angadi et al., 2015; Zaky et al., 2018; Sales et al., 2020; Kourek et al., 2021). The common downgrading items were the following: (a) item 2 and 3, 10 studies (71.4%) did not report the method used to generate the random allocation sequence and whether concealed allocation were conducted; (b) item 5, eight studies (57.1%) did not blind or did not describe blinding of the outcome assessors; (c) item 6, 11 studies (78.6%) did not specifically disclose the number of patients who started and completed the intervention period, and/or adherence was ≥ 85%; (d) item 7, 12 studies (85.7%) did not conduct intention-to-treat analysis; and (e) 11 studies (78.6%) did not carry out periodic assessments to maintain constant the relative intensity.

**TABLE 2:** Methodological quality assessment of included studies judged using TESTEX scale.

	Study quality					Study reporting							
Study	Item 1	Item 2	Item 3	Item 4	Item 5	Item 6	Item 7	Item 8	Item 9	Item 10	Item 11	Overall	Judgement
Anagnostakou et al. (2011)	1	0	1	1	1	0	0	1	1	1	1	8	Fair
Angadi et al. (2015)	1	0	0	1	1	0	0	0	1	1	1	5	Poor
Benda et al. (2015)	1	0	0	1	0	3	0	1	1	0	1	8	Fair
Currie et al. (2013)	1	0	0	1	0	0	0	1	1	0	1	5	Poor
Kourek et al. (2021)	1	0	0	1	0	0	0	1	1	0	1	5	Poor
Moholdt et al. (2012)	1	1	1	1	1	0	2	1	1	0	1	10	Good
Sales et al. (2020)	1	0	0	1	0	0	0	1	1	0	1	5	Poor
Suchy et al. (2014)/Gevaert et al. (2023)	1	0	0	1	0	0	0	1	1	1	1	6	Fair
Taylor et al. (2022)	1	0	0	1	1	0	2	1	1	0	1	8	Fair
Turri-Silva et al. (2021)	1	1	1	1	0	0	0	1	1	0	1	7	Fair
Valentino et al. (2022)	1	1	1	0	1	2	0	1	1	0	1	9	Good
Van Craenenbroeck et al. (2015)	1	0	0	1	1	0	0	1	1	1	1	7	Fair
Wisløff et al. (2007)	1	1	0	1	0	3	0	1	0	0	1	8	Fair
Zaky et al. (2018)	1	0	0	1	0	0	0	1	1	0	1	5	Poor

Item 1, eligibility criteria specified; Item 2, randomisation specified; Item 3, allocation concealment; Item 4, group similar at baseline; Item 5, blinding of assessors; Item 6, outcome measures assessed in 85% of patients; Item 7, intention-to-treat analysis; Item 8, between-group statistical comparisons reported; Item 9, point measures and measures of variability for all reported outcome measures; Item 10, relative exercise intensity remained constant; Item 11, exercise volume and energy expenditure.

### 3.4 Outcome measures

#### 3.4.1 Flow-mediated dilation

**FIGURE 2:**
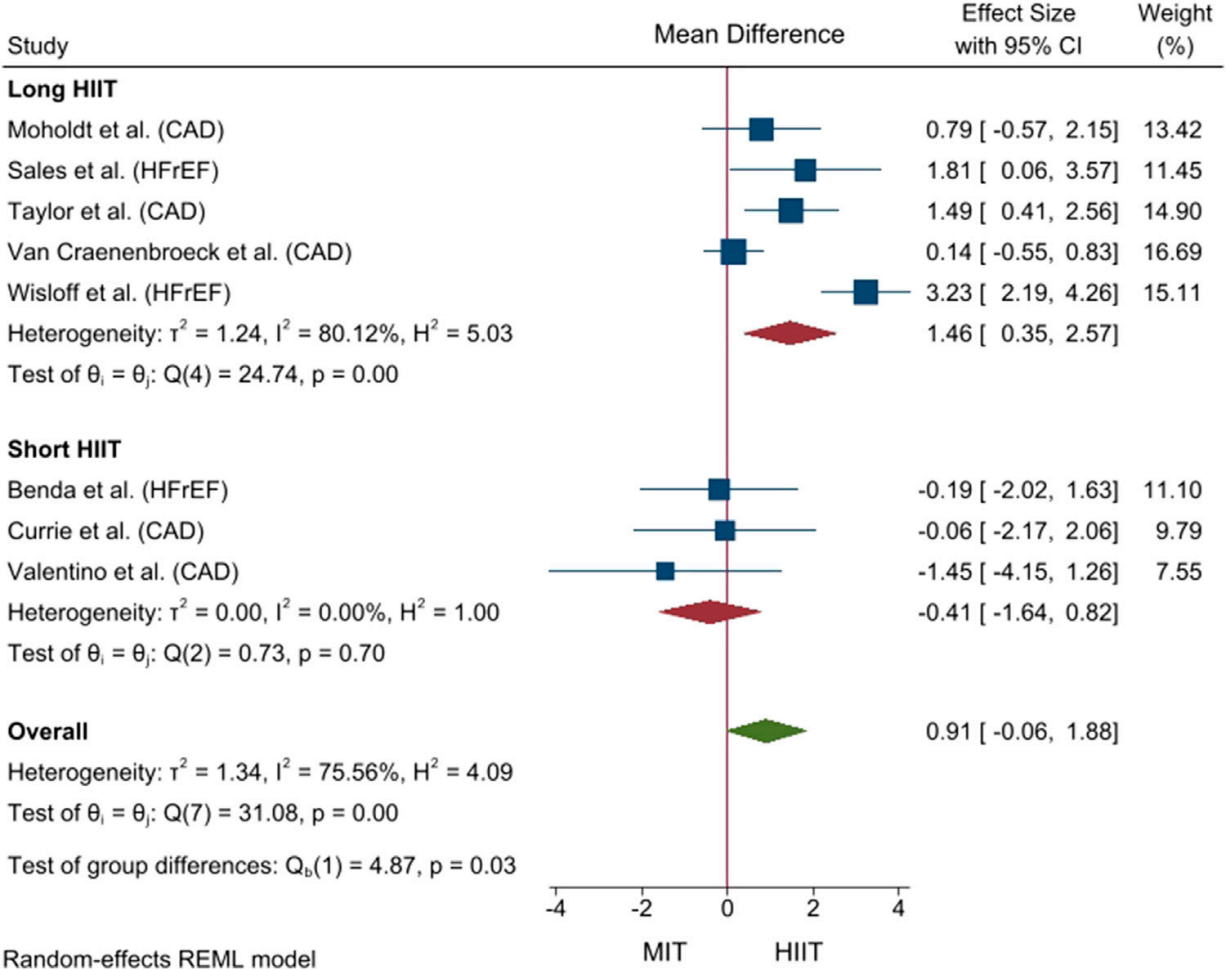
Forest plot of mean difference between high-intensity interval training and moderate intensity training based on the duration of high-intensity intervals for relative flow-mediated dilation. CAD, coronary artery disease; CI, confidence interval; HFrEF, heart failure with reduced ejection fraction; HIIT, high-intensity interval training; IV, inverse variance; MIT, moderate intensity training.

**FIGURE 3:**
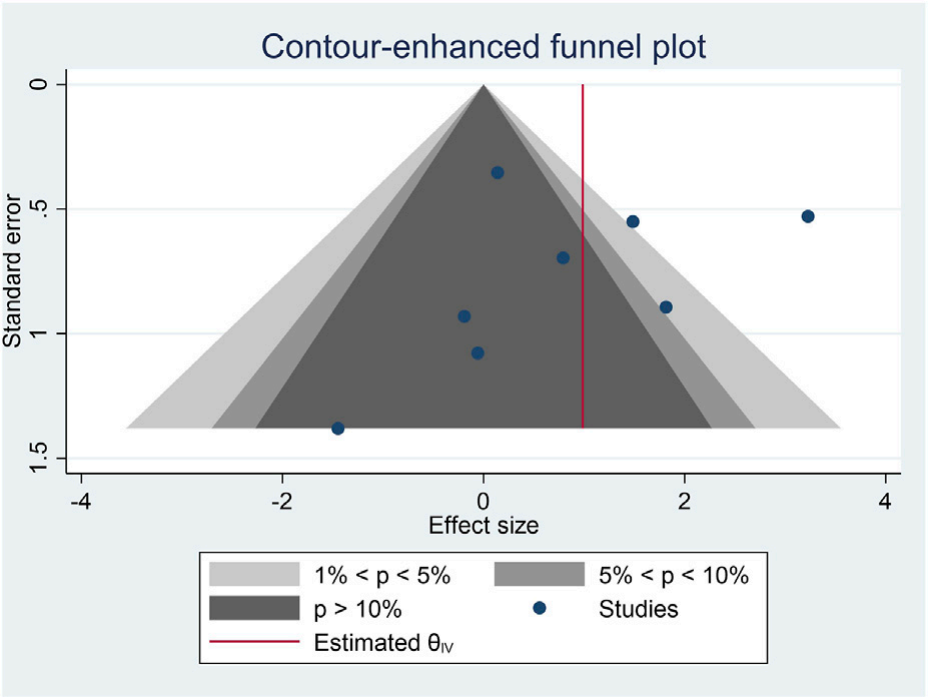
Contour-enhanced funnel plot for flow-mediated dilation.

Eleven studies compared the effect of HIIT and MIT on FMD. Two studies conducted with patients with HFpEF were excluded from meta-analyses (Suchy et al., 2014; Angadi et al., 2015). The non-pooled results are presented in Supplementary Table S3. Nine studies, with a total of 399 patients (HIIT = 187 and MIT = 212), were meta-analysed (Wisløff et al., 2007; Moholdt et al., 2012; Currie et al., 2013; Benda et al., 2015; Van Craenenbroeck et al., 2015; Zaky et al., 2018; Sales et al., 2020; Taylor et al., 2022; Valentino et al., 2022). The result showed that HIIT increases FMD to a higher degree than MIT in patients with CVD (i.e., CAD and HFrEF) (*p* = .030; MD_+_ = 1.24% [95% CI = 0.12, 2.36]). However, an influential study was found through sensitivity analyses (Zaky et al., 2018). After removing this study, there was no difference between both aerobic exercise methods for improving FMD (*p* = .066; MD_+_ = 0.91% [95% CI = −0.06, 1.88]). The heterogeneity test reached statistical significance (*p* < .001) with high inconsistency (*I* ^*2*^ = 75.6%). Therefore, the influence of potential moderator variables on the difference between the two aerobic exercise methods on FMD was analysed. The results are shown in Supplementary Table S4. Differences were found between studies which performed long HIIT and short HIIT (*p* = .027). Long HIIT enhanced relative FMD to a greater degree than MIT in patients with CVD (5 studies; MD_+_ = 1.46% [95% CI = 0.35, 2.57]) (Wisløff et al., 2007; Moholdt et al., 2012; Van Craenenbroeck et al., 2015; Sales et al., 2020; Taylor et al., 2022). In contrast, no difference was found between short HIIT and MIT (3 studies; MD_+_ = −0.41% [95% CI = −1.64, 0.82]) (Currie et al., 2013; Benda et al., 2015; Valentino et al., 2022) (Figure 2). No influence of the remaining analysed variables (i.e., pathology, only supervised exercise sessions, and intervention length) was found (*p* > .050). The Egger test did not reach statistical significance (*p* = .184) and the contour-enhanced funnel plot shows no asymmetry (Figure 3).

Anagnostakou et al. (2011) and Turri-Silva et al. (2021) tested whether HIIT was better than combined exercise and resistance exercise, respectively, for enhancing FMD (see Supplementary Table S3). Their findings will be discussed in the following section.

#### 3.4.2 Endothelial progenitor cells

Suchy et al. (2014) and Van Craenenbroeck et al. (2015) compared the effect of HIIT and MIT on EPCs, while Kourek et al. (2021) tested whether HIIT was better than combined exercise for improving EPCs. The low number of studies prevented us to conduct meta-analysis and their findings, which can be found in Supplementary Table S3, will be discussed below.

## 4 Discussion

The current systematic review and meta-analysis was undertaken to (a) compile the main characteristics of HIIT interventions aimed at improving endothelial function (i.e., brachial FMD and EPCs) in patients with CVD (i.e., CAD, HFrEF, and HFpEF), (b) investigate the effect of HIIT, compared to MIT and other exercise modalities (i.e., resistance exercise and combined exercise), on endothelial function in this population, and (c) analyse the influence of HIIT variables on the improvement of endothelial function. We will discuss the results of each of these objectives separately.

### 4.1 Characteristics of HIIT interventions

Consistent with the protocols of studies included in a previous review in healthy individuals (e.g., postmenopausal females) and patients with various pathologies (e.g., hypertension and CAD) (Ramos et al., 2015), most of the studies included in the current synthesis trained 3 days a week for 12 weeks. Moreover, they predominantly investigated the effect of long HIIT (e.g., four 4-min high-intensity intervals separated by active recovery periods), compared to MIT, on brachial FMD in patients with CAD or HFrEF. Therefore, the effect of other HIIT protocols (e.g., short intervals, passive recovery periods, and higher number of repetitions), as well as other exercise programmes (e.g., 2 days a week for 8 weeks), requires future study. In this regard, for instance, Wahl et al. (2014) reported that active recovery periods may increase angiogenic factors (e.g., vascular endothelial grow factors) more than passive recovery periods.

### 4.2 Effect of HIIT compared to MIT on brachial FMD

We found no difference between HIIT and MIT for improving brachial FMD in patients with CAD and HFrEF. It should be pointed out that we removed Zaky et al. (2018) from the pooled analysis because it was considered an influential study and could be interfering with the results. In this study, the authors disclosed that brachial FMD measurements were performed according to the guidelines (Thijssen et al., 2011). However, contrary to recommendations, the cuff was placed proximal (i.e., upper arm) to the brachial artery, which might explain their controversial findings. Our results conflict with previous research. Mattioni Maturana et al. (2021) and Ramos et al. (2015) reported that HIIT increased endothelial function to a greater extent than MIT. However, they analysed a different study population. Mattioni Maturana et al. (2021) included studies conducted with healthy and unhealthy individuals, while Ramos et al. (2015) included studies which enrolled participants with impaired endothelial function (e.g., type 2 diabetes or obesity).

Nonetheless, the results of our meta-analysis were inconsistent and, thus, we investigated the influence of HIIT variables on endothelial function. Interestingly, our subgroup analysis showed that long HIIT (e.g., four 4-min high-intensity intervals) increases brachial FMD to a greater degree than MIT in patients with CAD and HFrEF (MD_+_ = 1.46% [95% CI = 0.35, 2.57]). In contrast, there was no difference between short HIIT (i.e., ≤ 1 min) and MIT. In the same line, Klonizakis et al. (2014), Mitranun et al. (2014), and Bækkerud et al. (2016), which were included in the previous reviews (Ramos et al., 2015; Mattioni Maturana et al., 2021), found that short HIIT is not better than MIT in enhancing endothelial function in postmenopausal female patients with type 2 diabetes, and people with obesity, respectively. Nonetheless, Mattioni Maturana et al. (2021) did not include HIIT protocol as a covariate in their subgroup analyses, while Ramos et al. (2015) succinctly disclosed a possible influence of the HIIT protocol on the improvement of endothelial function. It should be noted that, in the current review, the results of studies comparing long HIIT and MIT were inconsistent (i.e., high heterogeneity) (see Figure 2) (Wisløff et al., 2007; Moholdt et al., 2012; Van Craenenbroeck et al., 2015; Sales et al., 2020; Taylor et al., 2022). Regarding the measurement of FMD, a post-deflation period of approximately 180 s is recommended (Black et al., 2008). However, Wisløff et al. (2007) used a shorter post-dilation timeframe of only 60 s. Moreover, we reported that most of the included studies had fair or poor methodological quality, which could affect their finding. These limitations should be considered when explaining the observed inconsistency in our pooled analysis, after taking into account the duration of the high-intensity bouts. Therefore, to the best of our knowledge, the current systematic review with meta-analysis is the first to provide evidence of the influence of the HIIT protocol (i.e., long vs. short HIIT) on the improvement of brachial FMD in patients with CVD (i.e., CAD and HFrEF).

Interestingly, Xu et al. (2014) and Matsuzawa et al. (2015) found that the adjusted relative risk of cardiovascular events per 1% improvement in FMD was 0.90 (95% CI = 0.88, 0.92) and 0.84 (95% CI = 0.79, 0.88), respectively, in patients with CVD, supporting the use of long HIIT in a clinical setting.

From a mechanistic point of view, aerobic exercise increases blood flow and shear stress, which stimulates NO production and thus improves endothelial function (Niebauer and Cooke, 1996). Based on previous evidence, HIIT is thought to induce a greater amount of shear stress than MIT on vascular walls of the exercising muscles, stimulating greater NO production (Davignon and Ganz, 2004; Tjønna et al., 2008). This was also demonstrated by Thijssen et al. (2009), who showed increased blood flow and shear stress with greater exercise intensity. Moreover, shear stress appears to be indirectly associated to biomarkers of endothelial dysfunction such as oxidative stress (Mohan et al., 2007) or inflammatory markers (Vion et al., 2013). Therefore, we hypothesise that short HIIT does not increase blood flow and shear stress more than MIT, which could explain the lack of differences between the two aerobic exercise methods in improving brachial FMD. However, future studies are needed to confirm and mechanistically explain our findings.

Two studies, Angadi et al. (2015) and Suchy et al. (2014), compared the effect of long HIIT (i.e., four 4-min high-intensity intervals) and MIT in improving brachial FMD in patients with HFpEF. Angadi et al. (2015) found a higher HIIT-induced effect on endothelial function after a 4-week CR programme (MD = 4.52% [95% CI = 1.35, 7.68]). On the contrary, Suchy et al. (2014) found that MIT is better than HIIT in increasing brachial FMD after a 12-week exercise programme (MD = −2.74% [95% CI = −4.33, −1.15]). However, it should be highlighted that those patients allocated to the MIT group carried out five sessions a week, while those allocated to the HIIT group performed three sessions a week. Ashor et al. (2015) reported that the greater the frequency of resistance exercise, the higher the improvement in FMD in healthy people. The greater training frequency in the MIT group in the Suchy et al. (2014) study may explain their controversial finding. Therefore, the low number of included studies and their controversial findings prevented us from drawing any firm conclusions about the difference between long HIIT and MIT in enhancing brachial FMD in patients with HFpEF, which should be addressed in future studies.

### 4.3 Effect of HIIT compared to other exercise modalities on brachial FMD

In the present systematic review, we included two studies which compared the effect of HIIT to other exercise modalities on brachial FMD. Due to insufficient data, we were unable to conduct a meta-analysis; however, we can extract the following information.

Firstly, Turri-Silva et al. (2021) compared long HIIT to resistance exercise and found no differences between groups (MD = −0.71% [95% CI = −4.37, 2.95). Therefore, it seems that resistance exercise alone was not superior to long HIIT regarding FMD improvement. However, the authors believe the study might have been underpowered due to the small sample size. Previous studies show similar results. Ashor et al. (2015) found that all exercise modalities (aerobic, resistance and combined exercise) enhanced FMD significantly in healthy adults and O'Brien et al. (2020) observed that whole-body resistance exercise did not improve FMD in the brachial or popliteal artery compared to short HIIT or MIT in healthy older adults. Nonetheless, when analysing these results, it is important to ask ourselves whether the resistance exercise regimen used in these studies elicited a sufficient stimulus to alter FMD. It is possible that a longer duration or higher training intensity is required to bring about physiological adaptations. In this regard, Ribeiro et al. (2017) discovered a dose-response relationship between the intensity of resistance exercise and the mobilisation of EPCs, with the highest exercise intensities (80% of one-repetition maximum) producing the largest increases in EPCs and angiogenic factors. In addition, Ashor et al. (2015) found a positive correlation between the frequency of resistance exercise and FMD.

Secondly, Anagnostakou et al. (2011) compared short HIIT to combined exercise (i.e., short HIIT and resistance exercise) for the improvement of brachial FMD and found significant differences in favour of the combined exercise group (MD = −4.66% [95% CI = −4.38, −1.94). Consequently, although resistance exercise alone was not superior to HIIT (Turri-Silva et al., 2021), these results suggest that combining both exercise modalities has an additional benefit on endothelial function. The mechanism by which resistance exercise improves endothelial function seems to be different from the increased shear stress induced-FMD improvement seen with aerobic exercise. It has been hypothesised that skeletal muscle contraction might produce transient ischemia which leads to reactive hyperaemia upon muscle relaxation with the subsequent increase in shear stress (Tinken et al., 2010). The existence of different pathways by which each exercise modality impacts endothelial function could account for the different effects of both exercise modalities on FMD or even the synergistic impact of combining the two. Therefore, in order to prescribe resistance exercise as a component of patients’ cardiac rehabilitation, further investigations are needed to ascertain the impact of brief, high-pressure shear stress patterns on FMD in patients with CVD and the influence of exercise variables (e.g., intensity, frequency and duration of sessions) (Williams et al., 2007).

### 4.4 Effect of HIIT on EPCs

Meta-analysis to determine the effect of HIIT, compared to MIT or other exercise modalities, on EPCs was not conducted due to the low number of studies included. The results of the existing studies are conflicting. Van Craenenbroeck et al. (2015) and Suchy et al. (2014) found no significant increase in the number of EPCs after training in patients with CAD and HFpEF, respectively. This was independent of the type of exercise protocol used (i.e., long HIIT or MIT). These results are surprising seeing as a potential mechanism that stimulates the mobilisation of EPCs from the bone marrow after exercise is shear stress (Tao et al., 2006; Boppart et al., 2015), leading to hypothesise that a greater shear stress (as occurs with HIIT protocols) would imply a greater mobilisation of EPCs. The authors point out that these results might indicate that EPCs are not critically involved in the training-induced improvement of endothelial function in patients with CVD. Conversely, Kourek et al. (2021) demonstrated that a single bout of maximum exercise was sufficient to enhance the mobilisation of EPCs in patients with HFrEF. Potential reasons for the differences between these studies are the lack of a widely accepted definition of EPCs, note the great variation between the measured EPCs’ phenotypes between studies (Supplementary Tables S2, S3) (Djohan et al., 2018). In addition, there is no gold standard measurement technique of EPCs. There are numerous described flow cytometric methods in the literature, with little to no agreement among them (Van Craenenbroeck et al., 2008; Huizer et al., 2017).

Regarding the comparison of the effect of HIIT and combined exercise (i.e., long HIIT and resistance exercise) in the mobilisation of EPCs, there were no differences between both groups (Kourek et al., 2021). Only one out of the five endothelial cell populations that were analysed (CD34^+^/CD45^-^/CD133^+^/VEGFR_2_) showed a higher number of resting EPCs with long HIIT compared to combined exercise. Therefore, the addition of resistance exercise did not enhance the mobilisation of EPCs compared to HIIT alone. These results are surprising seeing as the combination of HIIT and resistance exercise did have a positive impact over FMD, as commented in the paragraph above (Anagnostakou et al., 2011).

### 4.5 Strength and limitations

To the best of our knowledge, this review is the first to evaluate whether HIIT is better than MIT and other exercise modalities in improving endothelial function exclusively in patients with CVD. Furthermore, we have been the first to report the influence of the HIIT protocol (i.e., short vs. long HIIT) on the improvement of endothelial function. Nonetheless, there are some limitations that should be mentioned. First, the low number of studies which enrolled patients with HFpEF did not allow us to include them in the pooled analysis, and conclusions are limited to patients with CAD and HFrEF. Second, the influence of other HIIT variables (e.g., type of recovery) on the improvement of endothelial function was not investigated because most of the studies conducted the same HIIT protocol (e.g., four 4-min high-intensity bout interspersed with active recovery periods). Third, meta-analyses to determine whether HIIT enhances endothelial function to a greater extent than other exercise modalities were not performed. Forth, non-randomised studies were also included after careful evaluation of their methodological quality.

## 5 Conclusion and perspective

In summary, our results suggest that long HIIT (i.e., four 4-min high-intensity bouts separated by active recovery periods) is superior to MIT for improving brachial FMD in patients with CAD and HFrEF. On the contrary, there are no differences between short HIIT (length of high-intensity intervals ≤ 1 min) and MIT in increasing brachial FMD. Evidence for the effect of HIIT in patients with HFpEF is scarce. Future studies are required to test whether HIIT is better than resistance exercise or combined exercise for improving endothelial function (i.e., FMD and EPCs) in patients with CVD (i.e., CAD, HFrEF, and HFpEF). Therefore, cardiac rehabilitation programmes for patients with CVD aimed at enhancing endothelial function should centre on long HIIT protocols.

These results help shed some light on the effect of training variables (e.g., exercise modality) on endothelial function and could aid physicians to design the optimal cardiac rehabilitation program for patients with CVD, prioritising long HIIT protocols before MIT. Lastly, we cannot undermine the prognostic value this provides, since a 1% increase in FMD translates into an 8%—13% reduction in cardiovascular risk, improving the outcomes of patients suffering from CVD.

## Data Availability

The raw data supporting the conclusion of this article will be made available by the authors, without undue reservation.
