# Supplementary material for: Effects of high-intensity interval training on vascular function in patients with cardiovascular disease: a systematic review and meta-analysis
Source: Front Physiol. 2023 Jul 27;14:1196665. doi: 10.3389/fphys.2023.1196665 (PMC10413117; doi:10.3389/fphys.2023.1196665)
Supplement: Supplementary file 1 [file Table1.docx]

Supplementary Material

Effects of high-intensity interval training on vascular function in patients with cardiovascular disease: a systematic review and meta-analysis

Laura Fuertes-Kenneally, Carles Blasco-Peris, Antonio Casanova-Lizón, Sabina Baladzhaeva, Vicente Climent-Paya, José Manuel Sarabia, Agustín Manresa-Rocamora

*** Correspondence:** José Manuel Sarabia: [jsarabia@umh.es](mailto:jsarabia@umh.es)

**Database search terms**

(1) Patients. “Cardiovascular disease” OR “heart failure” OR “artery disease” OR atherosclerosis OR “coronary artery bypass” OR “percutaneous coronary intervention” OR angioplasty OR “ischaemic disease” OR “ischemic disease” OR “angina pectoris” OR “myocardial infraction” OR “myocardial infarction” OR “heart disease” OR myocardiopathy OR “acute coronary syndrome” OR atherectomy OR “heart attack” OR cardiovascular disease (MeSH Terms) OR acute coronary syndrome (MeSH Term) OR myocardial infarction (MeSH Term) OR heart failure (MeSH Term)

(2) Interventions. “Interval training” OR “interval exercise” OR “sprint training” OR “sprint exercise” OR HIIT OR “anaerobic training” OR “anaerobic exercise” OR “intermittent training” OR “intermittent exercise” OR high-intensity interval training (MeSH Term)

(3) #1 AND #2

(4) Outcomes. “flow-mediated dilat*” OR “endothelial-dependent dilat*” OR “endothelial-dependent vasodilat*” OR “endothelial function” OR FMD OR “endothelium-dependent dilat*” OR “endothelium-dependent vasodilat*” OR “vascular function” OR “endothelial dysfunction” OR “endothelium dysfunction” OR “brachial artery dilat*” OR “brachial artery vasodilat*” OR “nitric oxide” OR “reactive hyperaemia” OR “reactive hyperemia” OR “vascular endothelial growth factor” OR VEGF OR “endothelial progenitor cells” OR EPCs OR vascular function (MeSH Term)

(5) #3 AND #4

| **Item** | **Question** | **Additional Information** | **Scoring** |
| --- | --- | --- | --- |
| **Study Quality** | | | |
| 1 | Eligibility criteria specified | Eligibility criteria should be specified and fulfilled, and specific diagnostic test values should be provided for all participants | Yes (1) / No (0) |
| 2 | Randomisation specified | A description of the method used to allocate patients into the groups should be provided. Yes, if they are described and truly random (e.g., referring to a random number table, using a computer random number generator, coin tossing, shuffling card or envelopes, throwing dice, drawing of lots, and minimisation) | Yes (1) / No (0) |
| 3 | Allocation concealment | It should be stated if group allocation was concealed. Yes, if group allocation was concealed (e.g., central allocation, sequentially numbered drug containers of identical appearance, and sequentially numbered, opaque, sealed envelopes) | Yes (1) / No (0) |
| 4 | Groups similar at baseline | Baseline data of all participants who were allocated should be presented. Yes, if baseline data are separated by group allocation, presented, and no differences are apparent. | Yes (1) / No (0) |
| 5 | Blinding of assessors | Yes, if it is clearly stated that assessors of flow-mediated dilation are blinded to the allocation of the patients | Yes (1) / No (0) |

**Table S1.** Items and criteria used for the methodological quality assessment using TESTEX scale

| **Study Reporting** | | | |
| --- | --- | --- | --- |
| 6 | Assessment of outcome measures | The percentage of patients completing the study in the groups should be reported. No, if the number of patients at pre- and post-intervention is not clearly stated or adherence is < 85% (0 points). In contrast, 1 point if this information is clearly stated and adherence > 85%, 1 point if adverse events are reported, and 1 point if exercise attendance is reported | Yes (3) / No (0) |
| 7 | Intention-to-treat analysis | 1 point if all patients completed the intervention (no withdrawal) and 2 points if there are dropouts and intention-to-treat analysis has been used, regardless of the method used to carry out the imputation of the missing values. | Yes (2) / No (0) |
| 8 | Between-group statistical comparisons reported | Between-group comparisons should be reported for all included outcomes (i.e., FMD and/or EPCs). 1 point if between-group statistical comparisons are reported | Yes (1) / No (0) |
| 9 | Point measures and measures of variability for all reported outcome measures | Point estimates should be provided for all outcomes (i.e., FMD and/or EPCs), otherwise this could be deemed selective outcome reporting | Yes (1) / No (0) |
| 10 | Relative exercise intensity remained constant | A periodic assessment of exercise capacity should be conducted to maintain constant the relative intensity. 1 point if is performed | Yes (1) / No (0) |
| 11 | Exercise volume and energy expenditure reported | 1 point is training variables (i.e., session and programme length, session frequency, intensity, and modality) are clearly reported, regardless of the session (e.g., supervised, unsupervised, and group sessions) | Yes (1) / No (0) |

EPCs; endothelial progenitor cells; FMD, flow-mediated dilation

**Table S2.** Intervention and assessment characteristics

| **Study** | **Group** | **Intervention characteristics** | | **Assessment characteristics** |
| --- | --- | --- | --- | --- |
|  |  | **Setting; length; sessions a week** | **Session details** | **FMD: Cuff placement; occlusion length; occlusion pressure; post-deflation time window**  **EPCs: Method; EPC phenotypes; units** |
| Anagnostakou et al. (2011) | Short HIIT | Centre-based CR; 12 weeks;  3 sessions | Cycle ergometer; 40 min (session length); 30 s at 50% PPO at steep ramp test (105% PPO at cardiopulmonary exercise test) / 60 s (passive recovery) | FMD: distal; 300 s; 220 mmHg; 300 s (continuous measurements)  EPCs: not assessed |
|  | Combined exercise |  | Resistance exercise: 4 exercises; 3 sets × 10 – 12 reps / 30 s (recovery between sets) / quadriceps at 55 – 65% 2 RM (hamstrings – 1 kg), upper limbs at 10 RM  HIIT: Cycle ergometer; 20 min (session length) / The remaining characteristics were the same than in the HIIT group |  |
| Angadi et al. (2015) | Long HIIT | Centre-based CR; 4 weeks;  3 sessions | Treadmill; 10 min at 50% HR peak (WU) + 8 × 2 min at 80 – 85% HR peak / 2 min at 50% HR peak “week 1”; 4 × 4 min at 85 – 95% HR peak / 3 min at 50% HR peak “week 2 – 4” + 5 min at 50% HR peak (CD) | FMD: Based on guidelines (Correti et al., 2002)  EPCs: not assessed |
|  | MIT |  | Treadmill; 10 min at 50% HR peak (WU) + 15 min at 60% HR peak “week 1”; 30 min at 70% HR peak “week 2 – 4” + 5 min at 50% HR peak (CD) |  |
| Benda et al. (2015) | Short HIIT | Centre-based CR; 12 weeks;  2 sessions | Cycle ergometer; 10 min at 40% PPO (WU) + 10 × 1 min at 90% PPO (aiming 15 – 17 RPE) / 2.5 min at 30% PPO + 5 min at 30% PPO (CD) | FMD: distal; 300 s; 220 mmHg; 180 s (continuous measurements)  EPCs: not assessed |
|  | MIT |  | Cycle ergometer; 10 min at 40% PPO (WU) + 30 min at 60 – 75% PPO (aiming 12 – 14 RPE) + 5 min at 30% PPO (CD) |  |
| Currie et al. (2013) | Short HIIT | Centre- and home-based  CR; 12 weeks; 3 sessions | Cycle ergometer; 10 – 15 min light aerobic exercise (WU) + 10 × 1 min at 89% PPO (range, 80 – 104%) / 1 min at 10% PPO; 102% PPO “weeks 5 – 8”; 110% PPO for “weeks 9 – 12” + 10 – 15 min dynamic stretching (CD) | FMD: distal; 300 s; 200 mmHg; 180 s (continuous measurements)  EPCs: not assessed |
|  | MIT |  | Cycle ergometer; 10 – 15 min light aerobic exercise (WU) + 30 min at 58% PPO (range 51 – 65%); 40 min “weeks 5 – 8”; 50 min “weeks 9 – 12” + 10 – 15 min dynamic stretching (CD) |  |
| Kourek et al. (2021) | Long HIIT | Centre-based  CR; 12 weeks; 3 sessions | Cycle ergometer; 10 min at 45 – 50% VO_2_peak (WU) + 4 × 4 min at 80 – 105% VO_2_peak / 3 min at 50% VO_2_peak (31 min total length). Performance and balance exercises were also performed | FMD: Not assessed  EPCs: Flow cytometry;  CD34^+^/ CD45^-^/CD133^+^  CD34^+^/ CD45^-^/CD133^+^/VEGFR_2_  CD34^+^/CD133^-^/VEGFR_2_;  Cells / 10^6^ enucleated cells |
|  | Combined exercise |  | Resistance exercise: 2 – 3 sets × 10 – 12 reps at 60 – 75% 1RM / 1 min between sets (knee extension, knee flexion, and chest press)  HIIT: Same as HIIT group |  |

**Table S2.** Continued

| **Study (author)** | **Group** | **Intervention characteristics** | | **Assessment characteristics** |
| --- | --- | --- | --- | --- |
|  |  | **Setting; length; sessions a week** | **Sessions details** | **FMD: Cuff placement; occlusion length; occlusion pressure; post-deflation time window**  **EPC: Method; EPC phenotypes; units** |
| Moholdt et al. (2012) | Long HIIT | Centre- and home-based CR; 12 weeks; 3 sessions | Cycle ergometer; 8 min (WU) + 4 × 4 min 85 – 95% HR max / 3 min at 70% HR max + 5 min (CD) | FMD: proximal; 300 s; 250 mmHg; 60 s (no continuous measurement)  EPCs: not assessed |
|  | MIT |  | Aerobic exercise with music; 10 min (WU) + 35 min walking, jogging, lunges, and squats + 5 min stretching and relaxation exercises (CD) |  |
| Sales et al. (2020) | Long HIIT | Centre-based CR; 12 weeks;  3 sessions | Cycle ergometer; progressive work-to-recovery programme (1:1.5 “month 1”, 1:1 “month 2”, 1:0.67 “month 3”) at 5% above HR RCP; EE of 200 kcal/session | FMD: distal; 300 s; 220 mmHg; NR  EPCs: Not assessed |
|  | MIT |  | Cycle ergometer; Intensity between HR VT1 – HR RCP; EE of 200 kcal/session |  |
| Suchy et al. (2014) ^&^ | Long HIIT | Centre-based CR; 12 weeks;  3 sessions | Cycle ergometer; 10 min at 35 – 50% HRR (WU) + 4 × 4 min at 80 – 90% HRR / 3 min of active recovery | FMD: Information was not reported (abstract)  EPCs: Cytometry;  CD45^dim^/CD34^+^/VEGFR_2_^+^;  Cells / 10^6^ mononuclear cells |
|  | MIT | Centre- and home-based CR; 12 weeks; 5 sessions | Cycle ergometer; 40 min at 35 – 50% HRR (3 supervised sessions plus 2 sessions performed at home) |  |
| Taylor et al. (2022) ^&^ | Long HIIT | Centre- and home-based  CR; 4 weeks; 3 sessions | Aerobic exercise machines; 3 min < 11 RPE (WU) + 4 × 4 min at 15 – 18 RPE /  3 min at 11 – 13 RPE + 3 min < 11 RPE (CD) | FMD: Based on guidelines (Thijssen et al., 2010)  EPCs: Not assessed |
|  | MIT |  | Aerobic exercise machines ; 3 min < 11 RPE (WU) + 34 min at 11 – 13 RPE  + 3 min < 11 RPE (CD) |  |
| Turri-Silva et al. (2021) | Long HIIT | Centre-based CR; 12 weeks; 3 sessions | Cycle ergometer and treadmill (alternately); 10 min at ± 5% HR VT1 + 4 × 3 min at HR VT2 – 10% above HR VT2 / 4 min at 10% below HR VT1 – HR VT1 +  5 min (CD) | FMD: proximal; 300 s; 220 mmHg; 180 s (continuous measurement)  EPCs: not assessed |
|  | Resistance exercise |  | 10 min stretching and dynamic movements (WU) + circuit: 3 sets × 6 exercises (upper and lower limbs) at 60% 1 RM “month 1”, 70% 1 RM “month 2”, and 80% 1 RM “month 3”; 6 – 12 repetitions “weeks 1 – 2” and 15 – 20 repetitions “weeks 3 – 4” (within each month) + 5 min (CD) |  |

**Table S2.** Continued

| **Study** | **Group** | **Intervention characteristics** | | **Assessment characteristics** |
| --- | --- | --- | --- | --- |
|  |  | **Setting; length; sessions a week** | **Sessions details** | **FMD: Cuff placement; occlusion length; occlusion pressure; post-deflation time window**  **EPC: Method; EPC phenotypes; units** |
| Valentino et al. (2022) ^&^ | Short HIIT | Centre-based CR; 4 weeks; NR | Stairs; 10 min (WU) + 3 × 6 ascending of 12 steps (72 steps total) 14 – 15 RPE / 90 s of self-paced walking on flat ground + 5 min (CD) | FMD: distal; 300 s; 200 mmHg or 50 mmHg > SBP; 180 s (continuous measurement)  EPCs: not assessed |
|  | MIT |  | Cycle ergometer and treadmill; 10 min (WU) + 30 min 60 – 80% HRR + 5 min (CD) |  |
| Van Craenenbroeck et al. (2015) | Long HIIT | Centre-based  CR; 12 weeks; 3 sessions | Cycle ergometer; 10 min (WU) + 4 × 4 min at 90 – 95% HR max / 3 min at  50 – 70% HR max + 3 min (CD) | FMD: distal; NR; 200 mmHg or 60 mmHg > SBP; 180 s (continuous measurement)  EPCs: Cytometry;  CD34^+^/KDR^+^/CD45_dim_;  Cells / 10^6^ mononuclear cells |
|  | MIT |  | Cycle ergometer; 5 min (WU) + 37 min at 70 – 75% HR max + 5 min (CD) |  |
| Wisløff et al. (2007) | Long HIIT | Centre- and home-based CR; 12 weeks;  3 sessions | Centre: 2 s/w; uphill treadmill walking; 10 min at 60 – 70% HR peak (WU) + 4 × 4 min at 90 – 95% HR peak / 3 min at 50 – 70% HR peak + 3 min at 50 – 70% HR peak (CD); 38 min (total)  Home: 1 s/w; uphill outdoor walking; 4 × 4 min (intensity that made them to breathe heavily) | FMD: NR (Correti et al., 2002); 300 s; 250 mmHg; 60 s (no continuous measurements)  EPCs: Not assessed |
|  | MIT |  | Centre: 2 s/w; uphill treadmill walking; 47 min at 70 – 75% HR peak  Home: 1 s/w; uphill outdoor walking; 47 min without breathing heavily |  |
| Zaky et al. (2018) | Short  HIIT | Centre-based CR; 12 weeks;  3 sessions | Cycle ergometer; 5 min at 30% HR peak (WU) + 6 × 1 min at 90 – 95% HR peak / 4 min at 50 – 70% HR peak + 5 min at 30% HR peak (CD) | FMD: proximal; 300 s; 250 mmHg; 120 s (no continuous measurements)  EPCs: Not assessed |
|  | MIT |  | Cycle ergometer; 5 min at 30% HR peak (WU) + 30 min at 60 – 75% HR peak + 5 min at 30% HR peak (CD) |  |

CD, cool down; EE, energy expenditure; EPCs, endothelial progenitor cells; FMD, flow-mediated dilation; HIIT, high-intensity interval training; HR, heart rate; HR max, maximum heart rate; HRR, heart rate reserve; Long HIIT, long high-intensity interval training (> 1 min); MIT, moderate intensity training; NR, not reported; PPO, peak power output; RCP, respiratory compensation point; RM, repetition maximum; RPE, rate of perceived exertion; Short HIIT, short high-intensity interval training (≤ 1 min); s/w, sessions a week; VO_2_ peak, peak oxygen uptake; VT1, first ventilatory threshold; VT2, second ventilatory threshold; WU, warm-up

^&^ Refers to multistage studies

**Table S3.** Non pooled results

| - **Flow-mediated dilation** | | | | | |
| --- | --- | --- | --- | --- | --- |
| **Study** | **Pathology** | **Interval length** | **CG** | **n (HIIT/CG)** | **MD (95% CI)** |
| Angadi et al. (2015) | HFpEF | Long | MIT | 9/6 | 4.52 (1.35, 7.68) |
| Suchy et al. (2014) | HFpEF | Long | MIT | 15/18 | –2.74 (–4.33, –1.15) |
| Anagnostakou et al. (2011) | HFrEF | Short | CE | 14/14 | –4.66 (–7.38, –1.94) |
| Turri-Silva et al. (2021) | HFrEF and HFpEF | Long | RE | 5/6 | –0.71 (–4.37, 2.95) |
| - **Endothelial progenitor cells / phenotype** | | | | | |
| - **Study /** - **Phenotype** | - **Pathology** | - **Interval length** | **CG** | **n**  **(HIIT/CG)** | **MD (95% CI)** |
| Suchy et al. (2014)  CD45^dim^/CD34^+^/VEGFR_2_^+^ | HFpEF | Long | MIT | 40/46 | –8.92 (–22.3, 4.5) |
| Van Craenenbroeck et al. (2015)  CD34^+^/KDR^+^/CD45_dim_ | CAD | Long | MIT | 76/84 | –1.89 (–15.3, 11.5) |
| Kourek et al. (2021)  CD34^+^/CD45^-^/CD133^+^ | HFrEF | Long | CE | 21/23 | –2.67 (–21.2, 15.9) |
| Kourek et al. (2021)  CD34^+^/CD45^-^/CD133^+^/VEGFR_2_ | HFrEF | Long | CE | 21/23 | 1.96 (0.53, 3.4) |
| Kourek et al. (2021)  CD34^+^/CD133^-^/VEGFR_2_ | HFrEF | Long | CE | 21/23 | 10.2 (–2.8, 4.7) |

CAD, coronary artery disease; CE, combined exercise; CG, comparison group; CI, confidence interval; HIIT, high-intensity interval training; HFpEF, heart failure with preserved ejection fraction; HFrEF, heart failure with reduced ejection fraction; MD, mean difference; MIT, moderate intensity training; n, number of patients included to calculate mean difference; RE, resistance exercise

**Table S4.** Analyses of the influence of potential moderator variables on the difference of both aerobic exercise methods on relative flow-mediated dilation

| - **Categorical variables** | | | | **Test for subgroup differences** | |
| --- | --- | --- | --- | --- | --- |
| **Moderator** | **Category** | **K** | **MD (95% CI)** | ***Chi^2^*** | ***p*** |
| Pathology | HFrEF | 3 | 1.73 (–0.24, 3.70) | 1.33 | .249 |
|  | CAD | 5 | 0.49 (–0.26, 1.23) |  |  |
| HIIT protocol | Long HIIT | 5 | 1.46 (0.35, 2.57) | 4.87 | .027* |
|  | Short HIIT | 3 | –0.41 (–1.64, 0.82) |  |  |
| Only supervised exercise sessions | Yes | 4 | 0.25 (–0.59, 1.09) | 2.43 | .119 |
|  | No | 4 | 1.52 (0.17, 2.87) |  |  |
| Intervention length | Four weeks | 2 | 0.29 (–2.53, 3.12) | 0.22 | .637 |
|  | Twelve weeks | 6 | 1.02 (–0.12, 2.17) |  |  |

CAD, coronary artery disease; *Chi^2^*, chi-square statistic; CI*,* confidence interval; HFrEF, heart failure with reduced ejection fraction; HIIT, high-intensity interval training; *I^2^*, heterogeneity index; K*,* number of analysis units; MD, mean difference; *p*, probability level associated to *chi-squared* statistic; *, probability ≤ .050
